# Evaluation of a national program to distribute free face masks in Uganda: Evidence from Mbale District

**DOI:** 10.1371/journal.pone.0305574

**Published:** 2024-07-11

**Authors:** Aleksandra Jakubowski, Dennis Egger, Ronald Mulebeke, Pius Akankwasa, Allan Muruta, Noah Kiwanuka, Rhoda K. Wanyenze

**Affiliations:** 1 Department of Public Health and Health Sciences, Department of Economics, Northeastern University, Boston, United States of America; 2 Department of Economics, University of Oxford, Oxford, England; 3 School of Public Health, Makerere University, Kampala, Uganda; 4 Office of the Prime Minster, Kampala, Uganda; 5 Ministry of Health, Kampala, Uganda; Universite de Parakou, BENIN

## Abstract

**Background:**

COVID-19 posed a major threat to countries around the world, but many nations in sub-Saharan Africa avoided large-scale outbreaks. In Uganda, the government first enacted strict lockdowns but later focused on public health policies like masking and distancing. The government also embarked on an ambitious campaign to deliver a free face mask to all Ugandan citizens (approx. 30 million masks). We test whether mask distribution, and public education and encouragement of mask use by community health volunteers, affected mask behavior.

**Methods:**

We collected data about mask behavior before and after masks were distributed in the Mbale district of Uganda. Trained enumerators directly observed mask wearing in public places and asked about mask use via phone surveys. We compared observed and self-reported mask behavior before and after masks were distributed. We also tested whether training volunteers from randomly selected villages to educate the public about COVID-19 and masks affected behavior, attitudes, and knowledge among mask recipients.

**Results:**

We collected 6,381 direct observations of mask use at baseline (February 2021) and 19,855 observations at endline (April 2021). We conducted a listing of 9,410 households eligible for phone surveys and randomly selected 399 individuals (4.2%) at baseline and 640 (6.8%) at endline. Fewer than 1% of individuals were observed wearing masks at baseline: 0.9% were seen with a mask and 0.5% wore masks over mouth and nose. Mask wearing significantly increased at endline but remained low: 1.8% of people were observed with masks and 1.1% were seen wearing masks correctly after the distribution campaign. At the same time, a high proportion of people reported using masks: 63.0% of people reported using masks at baseline and 65.3% at endline when walking around their villages. When respondents were asked about mask use in public places, 94.7% reported using masks at baseline and 97.4% reported using masks at endline. We found no differences in knowledge, behavior, or attitudes among mask recipients in villages where volunteers were tasked with conveying information about COVID-19 and masks during distribution.

**Conclusion:**

Mask use remained low in Mbale district of Uganda during study observation period even after free masks were distributed. Encouraging new health behaviors may need to involve more intensive interventions that include reminders and address social norms.

## Background

COVID-19 posed a major threat to countries worldwide, but many nations in sub-Saharan Africa avoided large outbreaks. In Uganda, a country of approximately 46 million people, fewer than 4000 COVID-related deaths have been recorded throughout the pandemic [1]. Although it is likely that not every COVID death was captured in the official records, it is clear that the scale of the pandemic was smaller in Uganda relative to many other nations. One explanation for the relatively low spread of COVID-19 in Uganda and other sub-Saharan nations are the strict lockdown measures that were imposed early but were eventually eased due to the harsh social and economic consequences they had on the very populations they aimed to help [2]. Instead, governments began implementing public health requirements to physically distance, wear masks, and eventually vaccinate. The public health messaging about mask use was reinforced in Uganda with a national policy to distribute a free cloth face mask to every citizen of the country who was 6 years or older, or approximately 30 million people. Large-scale campaigns such as this one can garner public attention and media coverage, which may in turn communicate the importance of the public health recommendations; however, national distribution campaigns are also costly and logistically difficult to implement. Understanding the impact of the free mask distribution campaign in Uganda can provide important lessons for government responses in future public health emergencies.

During the COVID-19 pandemic, conflicting recommendations about masks were issued by health authorities over time. In the early stages of the pandemic, mask use was discouraged because public policy officials feared shortages of protective equipment for health professionals and worried about creating a false sense of security among the masked population. In June 2020, the World Health Organization reversed its guidance to recommend continuous mask wearing in all healthcare settings [3] and recommended mask use in public settings in December 2020 [4]. Subsequently, evidence has emerged to suggest that masks significantly reduce the spread of SARS-CoV-2 [5–8]. Given face masks’ low cost and relative ease of use, a key question facing health policymakers is whether populations are heeding the advice to wear masks, and what strategies are especially effective to encourage mask take-up. Mask distribution alone may be effective if lack of access to affordable masks is the main barrier, similar to what has been shown for other health goods [9,10]. Education and behavioral nudges may be needed if the barrier to adoption is households’ misperception of COVID-19 severity or mask effectiveness [11–13]. Distinguishing between these mechanisms may shed light on the mechanisms driving adoption of new health behaviors, providing lessons for future pandemics and public health emergencies.

Existing estimates of mask wearing in Uganda vary widely: 22%–71% in urban settings[14–16] and 70% to 95% in rural settings [17,18]. The wide range of estimates may be at least partially explained by social-desirability bias in self-reported data, or the tendency to overstate compliance with public health recommendations when social norms are clear [19]. Some studies attempted to address social-desirability bias by using “guilt-free” questions to assess mask wearing [20] but this strategy has limitations as it still relies on self-reports. Measuring social desirability bias of mask wearing is straightforward since the behavior is highly visible. In a related study in Kenya, 88% of survey respondents said they wear masks to public places but only 10% of people were observed with masks [21]. This gap illustrates that, although most people conform to the norm of mask-wearing in survey responses, adopting this new health behavior in practice remains a challenge. Objective measures of mask behavior using direct observations has the potential to significantly strengthen our understanding of how well public health rules were adhered to during COVID-19.

We partnered with the Office of the Prime Minister, the Ministry of Health, and the Mbale district officials to evaluate the national policy to distribute free masks in Uganda. Our study used phone surveys and direct observations of mask use from the Mbale district. We tested whether distribution of masks alone or distribution of masks paired with education about COVID-19/masks and a behavioral nudge to use masks affected mask wearing behavior, attitudes, and knowledge.

## Methods

This study measured mask use before and after mask distribution campaigns in the Mbale district of Uganda. We obtained non-causal estimates of the association between distribution campaigns and mask use by comparing directly observed and self-reported mask behavior before and after distribution took place. We then estimated the causal impact of pairing mask distribution with education and a behavioral nudge by randomly assigning which village health teams received additional training about masks and COVID-19. Baseline data were collected 04–21 February 2021, volunteer training was on 1–2 March 2021, masks were distributed 13–14 March 2021, and endline data were collected 11–30 April 2021. The volunteers were trained about COVID-19 and masks and were asked to repeat the information they learned to mask recipients. The study procedures are described in detail below and were registered with the American Economics Association registry (RCT ID: AEARCTR-0007844). The registry can be accessed at: https://www.socialscienceregistry.org/trials/7844.

### National mask distribution campaign

The government of Uganda aimed to distribute a free face mask to all citizens of the country who were 6 years or older. The government supplied masks to districts and left the last mile distribution logistics to the discretion of the district officials. As it is common with other health goods such as bed net distribution, districts officials typically leverage the extensive community health worker networks, locally called the village health teams (VHTs), to distribute public health goods to households; the same was done in the case of face masks. The distribution campaign started in June 2020, first prioritizing districts that were considered as highest risk for COVID-19 transmission: those on the borders with neighboring countries, highway districts where truck drivers moving from all corners of the country were possibly making contact with the local population, and densely populated districts. By November 2020, approximately half of the districts in Uganda had received masks, at which point the program slowed down due to low supply of masks and heightened political tensions ahead of the national elections. Following the elections in January 2021, the program resumed in February 2021, at which time face masks were delivered to Mbale district.

### Intervention

The Mbale district received masks in early March 2021 and distributed them on 13–14 March 2021. VHTs in all villages (n = 57 villages) received standard information leaflet about masks from the Ministry of Health, containing information on how to put on and clean a mask. Volunteers from a random set of villages (n = 36 villages) received an additional one-day training that included in-depth information about COVID-19, how masks work, why wearing a mask over mouth and nose is important, and a suggestion to hang the masks near a doorway to help them remember the masks on outings. Volunteers were asked to convey the information from their training to mask recipients during distribution.

### Setting

Mbale District is in the Eastern Region of Uganda near the border with Kenya and has a major trade route running through the district. The total population of Mbale is 465,000 people who live in 27 subcounties. We set to work in four subcounties (Busiu, Busiu Town Council, Bumasikye, Lukhonje) that have a total population of over 45,000 people who live in 173 villages. We randomly selected 90 villages to collect data. Due to implementation challenges we eliminated one of the subcounties, Busiu TC, from our study, which yielded a total sample of 57 villages where we conducted mask observations and phone surveys.

### Data

We make use of two data sources: i) phone surveys with randomly selected respondents, ii) direct observations of mask behavior in public spaces. Subjects were recruited for the study between 04 February and 30 April 2021.

### Phone surveys

The phone survey was designed to be population representative of the study area. Baseline phone surveys were conducted prior to the experimental start date (04–25 February 2021), targeting 7 randomly selected households in each village. Approximately 4 weeks after the experimental start (11–30 April 2021), we collected endline phone surveys, targeting 10 households per village. Both surveys captured information about household mask ownership, COVID-19 knowledge, and economic activity. Verbal informed consent was obtained from participants prior to conducting the phone survey.

### Mask observations

Mask use observations were conducted concurrently with the phone surveys. Enumerators observed public spaces from a safe distance and recorded mask use, type and features of mask wearing, and physical distancing of passersby. Each village was observed for at least three 60-minute time slots on different parts of the day. No human subjects data were collected as part of direct observation and the need for consent was waived by the ethics committee.

### Outcome variables

The main outcome variables of interest are whether participants self-disclosed mask use (phone survey data) and whether we observed mask use (observations data). Additional variables used in the analysis are related to respondents’ knowledge and attitudes about COVID-19 and mask use.

### Statistical models

We tested whether mask use differed before and after the mask distribution campaign. This estimate is not causal since we are not able to account for unobservable factors that could have affected mask use over the same time period. We fitted ordinary least squares regression in which the outcome variable was regressed on an indicator of whether the observation was from endline (baseline served as reference) controlling for a vector of individual characteristics of participants (sex, age, age-squared, marital status, whether household had any children under 5 years, and if the respondent did any non-agricultural work). Standard errors were clustered at the village level.

We then tested whether pairing the free mask distribution with VHT education campaigns and a behavioral nudge affected the outcome variables. We did this by fitting analysis of covariance (ANCOVA) regression models [22] in which the outcome variable was regressed on an indicator for the intervention, set to 1 if respondent lived in a village that was randomly selected to receive education and a behavioral nudge, or set to 0 in control villages that only received free masks and a standard MoH information sheet about masks. Models controlled for the baseline value of the outcome variable (set to the mean if missing) and the vector of individual characteristics specified above. Standard errors were clustered at the village level.

### Sensitivity analysis

We assess whether the intervention was delivered as intended by testing whether respondents in villages where community health volunteers received the one-day training recall receiving additional messages about COVID-19, mask effectiveness, and behavioral nudge (where to hang the mask to remember it). One subcounty was excluded from the analysis due to implementation issues. To maintain our power for statistical analysis, an additional set of villages were randomly selected for observation at endline. We test whether restricting the sample of direct observations to the set of villages observed at baseline and endline (n = 53) affects study findings.

### Ethics statement

Study procedures were approved by the Makerere University School of Medicine Research and Ethics Committee in Uganda (Protocol 875), the Ugandan National Council for Science and Technology (HS1124ES), and the University of California, Berkeley, Committee on Human Research in the United States (2020-09-13639). Informed consent was obtained verbally during phone interviews.

## Results

We recorded 6,381 direct observations of mask use at baseline and 19,855 direct observations of mask use at endline (Table 1). 63% of the observations were estimated to be conducted on males and nearly half (46%) were of people who are middle aged (26–45 years old category). Approximately 16.3% of observations at baseline and 12.9% at endline were based on interactive activities and another 8.9% on shopping or vending. 12.3% of observations at baseline and 16.0% of observations at endline were about commuting on public transportation, primarily boda bodas (for hire motorbikes). 67% of the mask observations at baseline and 60% at endline took place in crowded spaces.

**Table 1:** Description of study sample.

**Panel A: Mask Observations**	Baseline	Endline
	6,381	19,855
Number of villages in sample	54	87
Observed female sex	2368 (37.1%)	7,485 (37.7%)
Observed age categories		
18–25	1848 (29.0%)	5,506 (27.7%)
26–45	2960 (46.4%)	9,731 (49.0%)
46–60	1170 (18.3%)	3,599 (18.1%)
61+	403 (6.3%)	1,019 (5.1%)
Observed area is crowded / busy	4255 (66.7%)	11,887 (59.9%)
Observed activity		
Shopping / vending ^a^	569 (8.9%)	1,771 (8.9%)
Walking by / sitting / spending time alone	2447 (38.3%)	7,235 (36.4%)
Working	1377 (21.6%)	4,699 (23.7%)
Commuting	785 (12.3%)	3,181 (16.0%)
Talking / interacting with others	1042 (16.3%)	2,565 (12.9%)
Other	161 (2.5%)	404 (2.0%)
**Panel B: Phone Surveys**	Baseline	Endline
	N = 399	N = 640
Age	43.2 (17.9)	42.3 (17.6)
Female sex	188 (47.1%)	288 (45.0%)
Married or living with partner	271 (67.9%)	446 (69.7%)
*Education categories*		
None	20 (5.0%)	49 (7.7%)
Primary	221 (55.4%)	350 (54.7%)
Secondary	133 (33.3%)	201 (31.4%)
Above secondary	25 (6.3%)	40 (6.2%)
Agricultural work	324 (81.2%)	568 (88.8%)
Non-agricultural work (self-employed or salary)	149 (37.3%)	186 (29.1%)
Household size	6.7 (3.2)	6.1 (3.1)
Household has children under 5 years	235 (58.9%)	341 (53.3%)
Household has school-age children	363 (91.0%)	558 (87.2%)

^a^ Includes any activity that requires customer service interactions such as shops, barbers, restaurants, etc.

We conducted a listing of 9,410 households eligible for phone surveys and randomly selected 399 individuals (4.2%) at baseline and 640 individuals (6.8%) at endline for the survey. The average age of respondents was 43 years at baseline and 42 years at endline and about half were female (47% at baseline and 45% at endline). More than 60% of people had a basic education level of either no formal schooling or primary school and over 80% of respondents were farmers. The vast majority of respondents’ households included school-age children.

**Fig 1:**
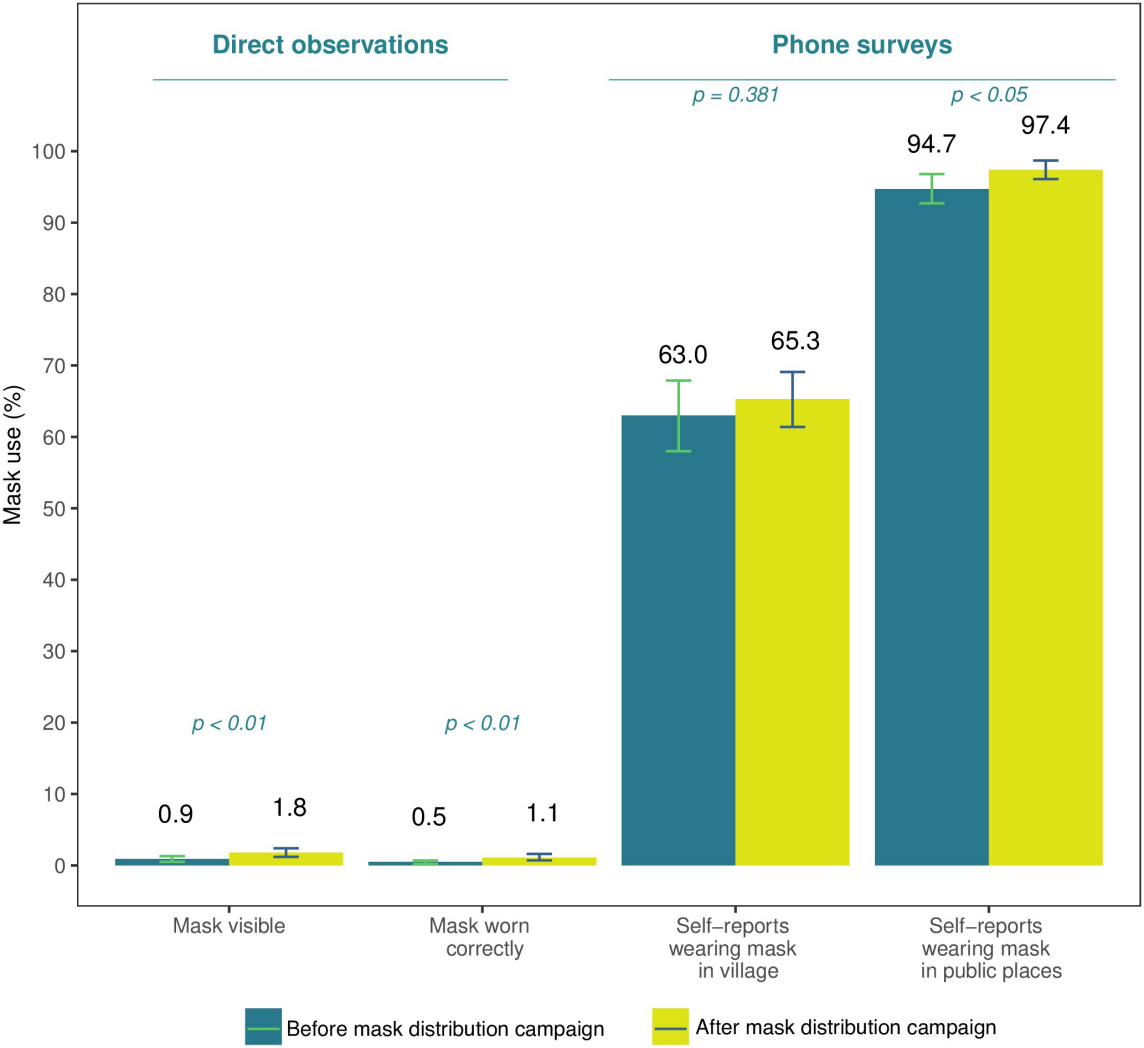
Mask behavior before and after free mask distribution. **Notes:** Based on a sample of 6,381 direct observations at baseline, 19,855 observations at endline, 399 phone surveys at baseline and 640 phone surveys at endline. Mask visible means the individual was observed with a mask but was not wearing it over mouth and nose. Mask worn correctly means the observed individual wore mask over mouth and nose. Phone survey respondents were asked about mask use in the last 7 days in any public places and when walking around their village.

Directly observed mask use was very low in the study region between February and April 2021 (Fig 1).

Fewer than 1% of observed people had masks with them at baseline and 1.9% of observed people had a mask at endline (difference: 0.9 percentage points, 95% CI [0.4–1.5], p<0.01). Even fewer people were seen wearing masks correctly over their mouth and nose: 0.5% at baseline and 1.1% at endline (difference: 0.6 percentage points, 95% CI [0.2–0.9], p<0.01). Study findings were very similar when we restricted the sample to direct observations repeated in the same set of villages (S1 and S2 Tables in S1 Appendix and S1 Fig in S1 Appendix).

**Fig 2:**
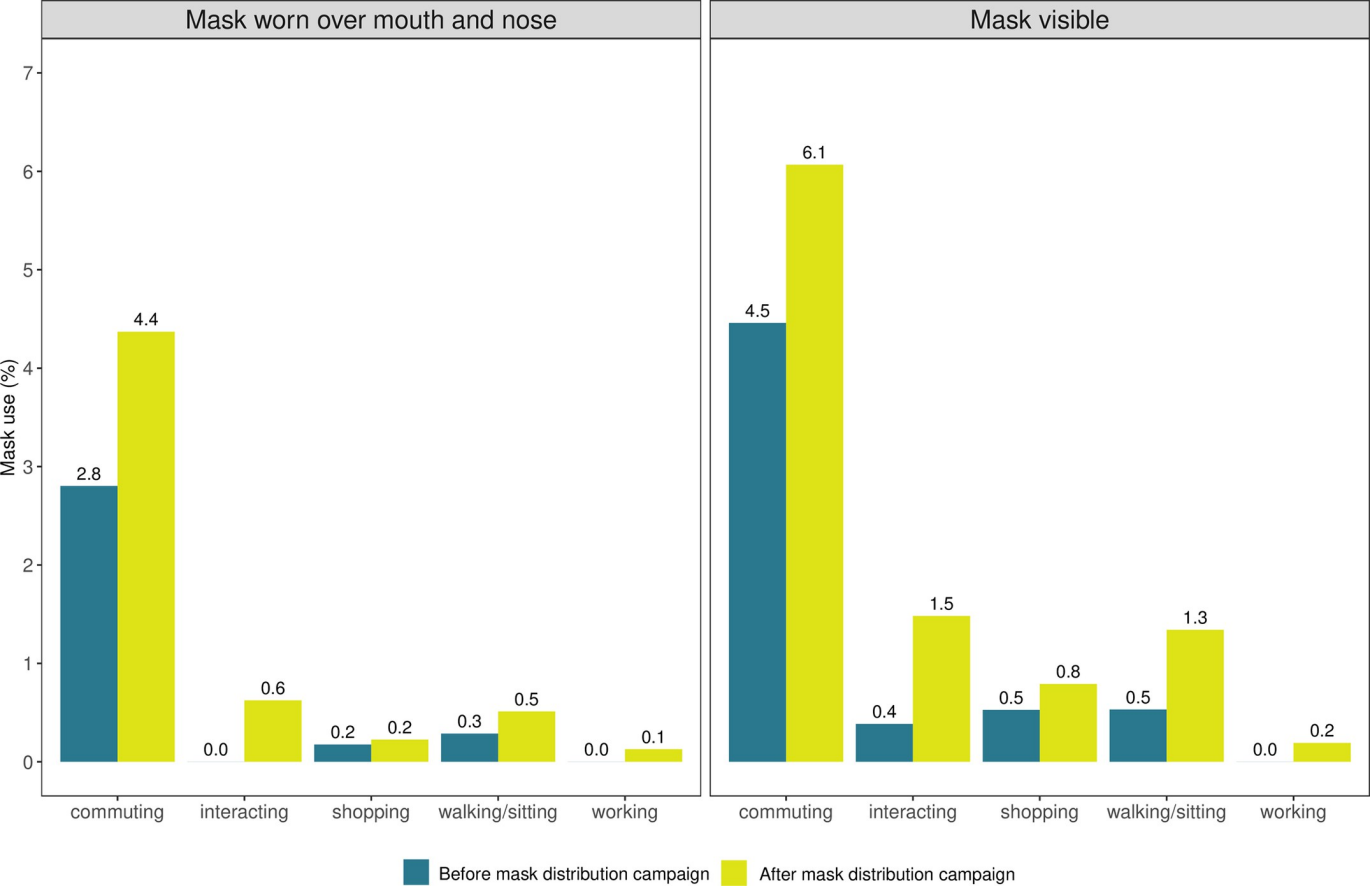
Observed mask behavior before and after free mask distribution by activity type. **Notes:** Based on a sample of 6,381 direct observations at baseline, 19,855 observations at endline, Mask visible means the individual was observed with a mask but was not wearing it over mouth and nose. Mask worn correctly means the observed individual wore mask over mouth and nose. “Commuting” category includes people riding on buses or boda-bodas, “interacting” category includes people who are engaged in conversations, “shopping” category includes people engaged in transactions, “walking/sitting” category includes people spending time in public places, “working” category includes people engaged in work activities.

We found variation in observed mask use when we stratified direct observations by activity type (Fig 2).

Mask use was highest among commuters. Before mask distribution 2.8% of commuters were observed wearing a mask correctly and 4.5% of commuters had a mask with them. After the intervention, mask use among commuters increased to 6.1% of people seen with a mask and 4.4% seen wearing the mask correctly over mouth and nose. Mask use was very low among all other activity types, including people who were seen talking or interacting with each other.

Most phone survey respondents reported using masks in public places in the past 7 days: 94.7% at baseline and 97.4% at endline (Table 2. Adjusted difference: 2.9 pp increase, 95% CI 0.6–5.3). When asked about mask wearing in specific situations, respondents reported wearing masks most frequently at religious gatherings (89.9% at baseline and 93.4% at endline) and on public transportation (84.7% at baseline and 87.9% at endline). Mask use at work was the lowest (52.8% at baseline and 58.6% at endline) followed by visiting another household (56.1% at baseline and 64.3% at endline). Self-reported mask use increased significantly after mask distribution campaigns (Table 2 Panel B), with the largest increases reported by participants who visited market centers (10.2 percentage points increase, 95% CI 4.3–16.1) and when visiting another household (9.2 percentage point increase, 95% CI 3.0–15.4).

**Table 2:** Observed and self-reported mask behavior before and after mask distribution.

**Panel A: Mask Observations**	Baseline (N = 6,381)	Endline (N = 19,855)	Adjusted difference	95% CI
	N (%)	N (%)		
Observed having a mask	57 (0.9%)	373 (1.9%)	0.9	[0.4–1.5]
Observed wearing a mask	30 (0.5%)	218 (1.1%)	0.6	[0.2–0.9]
**Panel B: Phone Surveys**	Baseline (N = 399)	Endline (N = 640)	Adjusted difference	95% CI
	N (%)	N (%)		
*Self-reported wearing mask*:				
public places ^a^	360 (94.7%)	606 (97.4%)	2.9	[0.6–5.3]
market centers ^b^	279 (70.8%)	504 (79.4%)	10.2	[4.3–16.1]
religious gatherings ^b^	356 (89.9%)	595 (93.4%)	4.1	[1.2–7.0]
public transportation ^b^	337 (84.7%)	561 (87.9%)	4.2	[0.0–8.5]
village store ^b^	250 (63.0%)	415 (65.3%)	3.1	[-3.2–9.3]
another household ^b^	224 (56.1%)	410 (64.3%)	9.2	[3.0–15.4]
at work ^b^	207 (52.8%)	366 (58.6%)	7.7	[1.5–13.9]

**Notes:** Direct observation models were adjusted for observed age and sex of observed person, whether the observed place was busy/crowded, and activity type. Phone survey data were adjusted for sex, age, age-squared, marital status, whether household had any children under 5 years, and if the respondent did any non-agricultural work.

^a^ Reported wearing a mask when going to public places in past 7 days. Includes any activity that requires customer service interactions such as shops, barbers, restaurants, etc.

^b^ Reports always or sometimes wearing a mask in specific situations.

We found no statistically significant differences in mask behavior, attitudes, or knowledge in villages where volunteers who were trained about masks and COVID-19 were tasked to educate the public about these issues (Table 3). About half of survey respondents (50.6%) knew that COVID-19 could be spread through breath. 97.5% of respondents said that face masks reduce the spread of COVID-19 and 77% believed that people in Uganda were at risk of COVID-19 infection at the time of the survey. Among the most cited new behaviors since the start of COVID were mask wearing (86.3%) and washing hands more frequently (93.0%). Only 32% of respondents said they avoided large group gathering since the start of the pandemic. About half of the respondents reported some flu-like symptoms in the past 30 days and 36% said they lost time from work or usual activities due to illness. Many respondents reported experiencing mental health symptoms: 56.1% felt anxious, 61.1% felt depressed, and 51.9% felt lonely. Although we did not have objective observations of mask distribution, we collected self-reported data about the topics of discussion covered when participants received mask. We found that participant who lived in villages randomized to receive volunteer training were 5.6 pp (95% CI -0.5, 11.7) more likely receive information about COVID-19, 6.7 pp (95% CI 1.1, 12.4) more likely to receive information about mask effectiveness, and 7.8 pp (95% CI 2.0, 13.6) more likely to receive a nudge to hang their mask by the door to remember to bring it out with them (S3 Table in S1 Appendix).

**Table 3:** Impact of pairing education about COVID19 and masks with free mask distribution.

**Panel A: Mask Observations**	Mean in control villages	Impact of volunteer training	95% CI
Observed having a mask	1.9	-0.1	[-1.2–1.1]
Observed wearing a mask	1.3	-0.3	[-1.2–0.6]
**Panel B: Phone Surveys**			
*Self-reported wearing mask to locations*:			
public place ^a^	96.5%	1.6	[-1.2–4.3]
market centers ^b^	76.4%	5.7	[-0.4–11.8]
religious gatherings ^b^	92.1%	2.7	[-0.9–6.2]
public transportation ^b^	86.9%	2.8	[-1.6–7.2]
village store ^b^	65.3%	0.6	[-7.8–8.9]
another household ^b^	65.0%	-1.6	[-8.5–5.3]
at work ^b^	63.1%	-6.1	[-13.5–1.4]
*Says COVID-19 spreads through*:			
surfaces	43.1%	-0.6	[-8.9–7.8]
droplets	79.9%	-4.4	[-9.7–0.9]
breath	50.6%	2.7	[-6.7–12.0]
*Beliefs about COVID-19 / masks*			
People in Uganda are at risk of infection	77.0%	1.9	[-4.7–8.5]
Masks reduce spread of COVID-19	97.5%	0.7	[-1.6–2.9]
*Behavior changes due to COVID*			
Stayed home more	39.2%	3.5	[-4.2–11.2]
Wore mask	86.3%	-5.5	[-12.1–1.1]
Avoided groups and gatherings (church, family etc)	32.6%	-3.1	[-10.0–3.8]
Washed hands more	93.0%	-0.8	[-4.9–3.3]
Avoided handshakes	33.0%	-4.2	[-11.3–3.0]
*Social distancing*			
Visited religious gathering outside village	34.0%	-1.8	[-11.5–7.9]
*Physical health*			
Experienced any COVID-19 symptoms ^c^	54.0%	-2.5	[-11.2–6.2]
Lost time from usual activities due to illness	36.4%	-3.0	[-12.5–6.5]
*Mental health*			
Felt nervous, anxious, on edge	56.1%	-3.8	[-11.2–3.5]
Felt depressed	61.1%	-1.1	[-9.2–7.0]
Felt lonely	51.9%	1.8	[-6.6–10.2]

**Notes**: Impact of intervention was estimated using endline data (11,657 direct observations and 640 phone interviews) and represents change from control mean. Models controlled for characteristics of individuals (observed age and female sex for observed data and age, age-squared, sex, education level, marital status, whether individual did any non-agricultural work in last 2 weeks and if the respondent lived in a household with children under age five). Standard errors clustered at village level.

^a^ Reported wearing a mask when going to public places in past 7 days. Includes any activity that requires customer service interactions such as shops, barbers, restaurants, etc.

^b^ Reports always or sometimes wearing a mask in specific situations

^c^ Experienced any of the following symptoms: Fever, persistent cough; always feeling tired; muscle pain (myalgia); headache; diarrhea, nausea or vomiting; difficulty breathing or chest pain; runny nose; sore throat; pneumonia, loss smell or taste.

## Discussion

Our study evaluated the effect of distributing free face masks alone and distributing free masks paired with education about masks and COVID-19 in the Mbale district, eastern Uganda, during a large-scale national campaign to deliver free masks to citizens of Uganda. We measured mask use through directly observing people in public places and through self-reports via phone surveys. We found very limited compliance with mask rules using direct observations and high compliance with masks mandates using self-reported data. At baseline, the proportion of mask use was very low: 0.5% of observed people were seen wearing a mask over their mouth and nose and 0.9% were seen carrying a mask but not wearing it at time of observation. Though mask use significantly increased by endline, it remained low with 1.1% seen using a mask correctly and 1.9% having a mask with them; an increase of only 0.6 percentage points and 0.9 percentage points, respectively. This suggests that lack of access to masks was not the main reason for low adoption of mask use in the region.

Among the main findings from this study is the vast discrepancy in self-reported vs observed mask use. Very few people who we observed in public wore masks, but a majority reported always or sometimes wearing masks to public places. This finding is in line with previous research in Kenya, where a similar discrepancy was found [21]. Other studies in Uganda which used survey data estimated mask wearing at 22%–71% in urban settings[14–16] and 70% to 95% in rural settings [17,18]. Our study underscores the large social desirability bias that exists in survey data on mask use, which may have significantly skewed the prior statistics on mask use upwards [19]. If mask use policies are enacted in future pandemics, researchers and public health officials need to incorporate direct observations of mask use to obtain reliable and unbiased statistics on compliance.

Our study tested whether training community health volunteers, who were tasked with the last mile distribution, led to increased use of the masks that were distributed through the national campaign. We randomly selected some villages where we trained the volunteers about COVID-19, the proper use of masks, and the importance of masks. The trained volunteers were then directed to relay this information to the mask recipients. We found no evidence that training volunteers to educate mask recipients about COVID-19 and masks affected survey respondents’ knowledge or attitudes about masks. This suggests that shifting public health behaviors and social norms may be difficult, and more intensive, targeted interventions may be necessary. For example, interventions that repeatedly remind people to wear masks, involve role models, or directly address the social norms around mask wearing may be especially effective.

Our study was subject to several limitations. Since mask distribution was not randomized, the increase in mask use from before to after masks were distributed is a non-causal estimate. In other words, multiple issues not related to mask distribution may have affected mask use over the same time period, which could explain the change in mask use observed in the study. Although the volunteer training intervention was randomized, our study lacked the capacity to observe how well the volunteers conveyed the information from the training to mask recipients. It is possible that information was not conveyed as it was intended (or at all) when volunteers visited households to deliver masks. We are reassured by self-reported data that indicates that participants who lived in villages randomized to receive volunteer training were significantly more likely to receive information about COVID-19, mask effectiveness, and advice on where to hang the mask to remember it. We find strong evidence of social desirability bias about mask use in the phone survey; similar bias may have affected responses to other questions in the survey and our ability to measure bias in other responses is more limited. Our study was timed to coincide with when the national mask distribution campaign reached the Mbale district rather than when COVID-19 surges occurred. Coincidentally, COVID-19 cases were quite low in Uganda in February—April 2021 when data were collected and masks were distributed. Our study is a snapshot of mask wearing behavior in one district and for a limited amount of time; future research should explore whether heterogeneity in COVID-19 severity and the norms around mask wearing—which may shift at times of surges—affect mask wearing behavior and attitudes.

When pandemics occur, policy officials are faced with finding the most effective ways to protect their citizens. Our study gives insight about where investments in interventions may yield the largest impact. We find that distributing free face masks alone did not meaningfully increase mask use. Training community health volunteers about COVID-19 and masks also did not translate to greater knowledge or shift in attitudes among the mask recipients. Behavioral interventions may be needed to affect change in mask usage, and focus should be on finding cost-effective solutions. Importantly, incorporating direct observations of mask use and other publicly observable health behaviors is needed to ensure that the measures of compliance with public health policies are unbiased.

## Supporting information

S1 Appendix S1 Table. Description of mask observations when sample is restricted to 53 villages that have data before and after mask distribution. S1 Fig. Analysis of mask use repeated on sample restricted to 53 villages where observations were conducted before and after mask distribution. S3 Table. Self-reported data about mask distribution.
(PDF)
