# Supplementary material for: Evaluation of a national program to distribute free face masks in Uganda: Evidence from Mbale District
Source: PLoS One. 2024 Jul 11;19(7):e0305574. doi: 10.1371/journal.pone.0305574 (PMC11238958; doi:10.1371/journal.pone.0305574)
Supplement: S1 Appendix — S1 Table. Description of mask observations when sample is restricted to 53 villages that have data before and after mask distribution. S1 Fig. Analysis of mask use repeated on sample restricted to 53 villages where observations were conducted before and after mask distribution. S3 Table. Self-reported data about mask distribution. (PDF) [file pone.0305574.s001.pdf]

Supplementary Appendix

Evaluation of a national program to distribute free face masks for COVID-19 prevention in Uganda: evidence from Mbale District

Aleksandra Jakubowski, Dennis Egger, Ronald Mulebeke, Pius Akankwasa, Allan Muruta, Noah Kiwanuka, Rhoda Wanyenze

Section 1. Analysis repeated on a dataset restricted to 53 study villages with data before and after mask distribution

S1 Table. Description of study sample when sample is restricted to 53 villages

**Panel A: Mask Observations**	Baseline	Endline
	6,284	11,657
Number of villages in sample	53	53
Observed female sex	2,334 (37.1%)	4,403 (37.8%)
Observed age categories		
18-25	1,835 (29.2%)	3,248 (27.9%)
26-45	2,915 (46.4%)	5,707 (49.0%)
46-60	1,138 (18.1%)	2,111 (18.1%)
61+	396 (6.3%)	591 (5.1%)
Observed area is crowded / busy	4,255 (67.7%)	6,803 (58.4%)
Observed activity		
Shopping / vending [a]	562 (8.9%)	1030 (8.8%)
Walking by / sitting / spending time alone	2,414 (38.4%)	4,188 (35.9%)
Working	1,360 (21.6%)	2,893 (24.8%)
Commuting	774 (12.3%)	1,889 (16.2%)
Talking / interacting with others	1,017 (16.2%)	1,433 (12.3%)
Other	157 (2.5%)	224 (1.9%)

S2 Table. Observed and self-reported mask behavior before and after mask distribution

**Panel A: Mask Observations**	Baseline (N=6,284)	Endline (N=11,657)	Adjusted difference	95% CI
	N (%)	N (%)		
Observed having a mask	57 (0.9%)	212 (1.8%)	0.8	[0.2 - 1.3]
Observed wearing a mask	30 (0.5%)	130 (1.1%)	0.5	[0.1 - 1.0]

**Panel B: Phone Surveys**	Baseline (N=399)	Endline (N=640)	Adjusted difference	95% CI
	N (%)	N (%)		
Self-reported wearing mask to locations: [a]				
public place	360 (94.7%)	606 (97.4%)	2.9	[0.6 - 5.3]
market centers	279 (70.8%)	504 (79.4%)	10.2	[4.3 - 16.1]
religious gatherings	356 (89.9%)	595 (93.4%)	4.1	[1.2 - 7.0]
public transportation	337 (84.7%)	561 (87.9%)	4.2	[0.0 - 8.5]
village store	250 (63.0%)	415 (65.3%)	3.1	[-3.2 - 9.3]
another household	224 (56.1%)	410 (64.3%)	9.2	[3.0 - 15.4]
at work	207 (52.8%)	366 (58.6%)	7.7	[1.5 - 13.9]

Notes: Direct observation models were adjusted for observed age and sex of observed person, whether the observed place was busy/crowded, and activity type. Phone survey data were adjusted for sex, age, age-squared, marital status, whether household had any children under 5 years, and if the respondent did any non-agricultural work.

[a] Includes any activity that requires customer service interactions such as shops, barbers, restaurants, etc.

Evaluation of a national program to distribute free face masks for COVID-19 prevention in Uganda: evidence from Mbale District

Aleksandra Jakubowski, Dennis Egger, Ronald Mulebeke, Pius Akankwasa, Allan Muruta, Noah Kiwanuka, Rhoda Wanyenze

S1 Fig. Analysis of mask use repeated on sample restricted to 53 villages

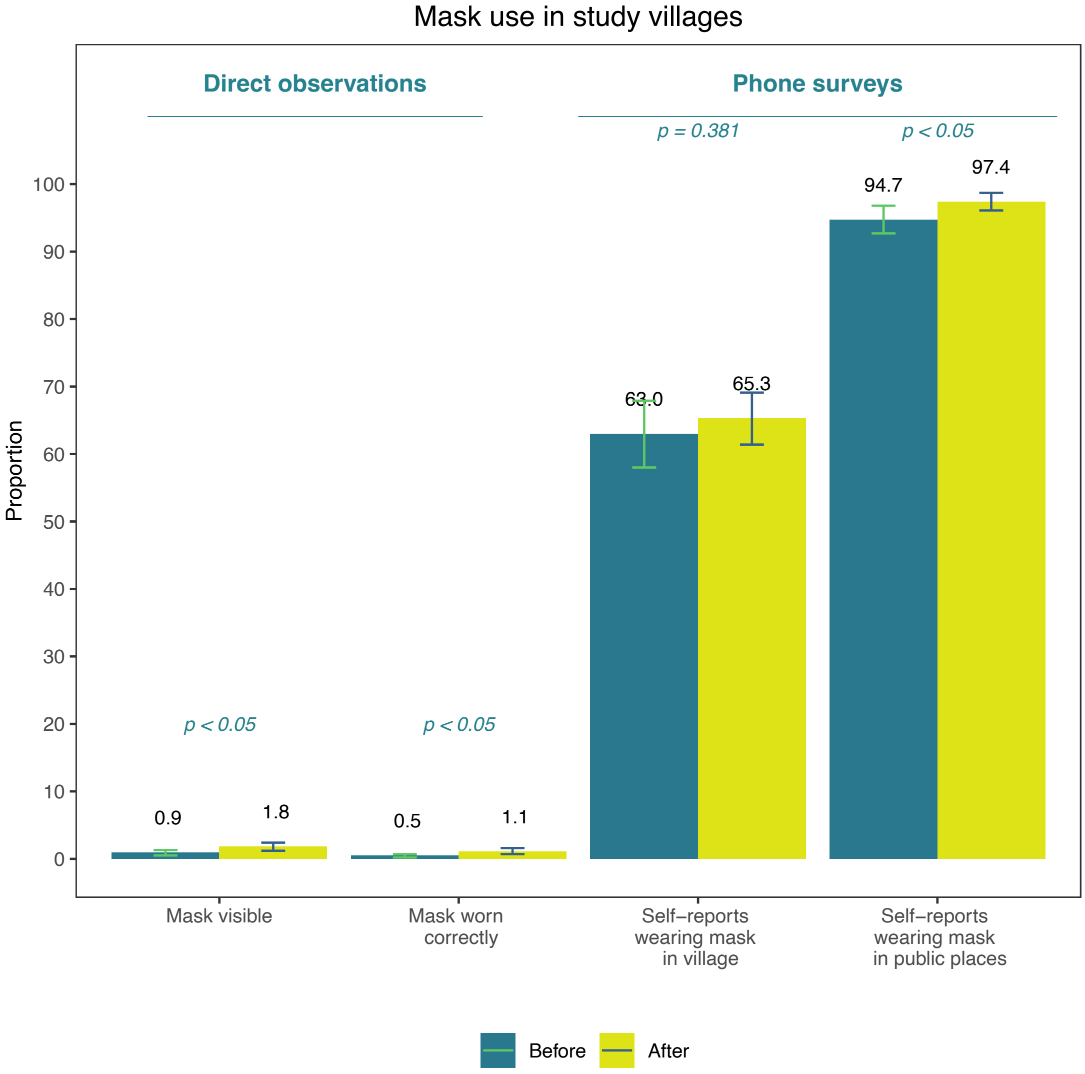

Notes: Based on a sample of 6284 direct observations at baseline, 11657 observations at endline, 399 phone surveys at baseline and 640 phone surveys at endline. Mask visible means the individual was observed with a mask but was not wearing it over mouth and nose. Mask worn correctly means the observed individual wore mask over mouth and nose. Phone survey respondents were asked about mask use in the last 7 days in any public places and when walking around their village.

Section 2. Phone survey respondents were asked about mask distribution, including whether they received free masks as part of the distribution campaign and what information the community health volunteer may have relayed during the distribution.

S3 Table. Self-reported data about mask distribution

	Mean in control villages	Impact of volunteer training	95% CI
Household received free masks distributed by the government	95.0%	1.0	[-2.9 - 4.9]
Volunteers shared information about COVID-19	86.1 %	5.6	[-0.5 - 11.7]
Volunteers instructed how to put on a mask correctly	85.1 %	7.0	[0.9 - 13.2]
Volunteers advised to maintain physical distance	84.2 %	7.3	[1.4 - 13.3]
Volunteers advised to wash hands frequently	85.1 %	6.5	[0.4 - 12.7]
Volunteers shared information on mask effectiveness	85.6 %	6.7	[1.1 - 12.4]
Volunteers suggested where to hang mask to remember it	83.7 %	7.8	[2.0 - 13.6]
